# Precision Medicine in Rural Settings: Patient Perspectives on Clinician-Ordered Genetic Testing

**DOI:** 10.21203/rs.3.rs-9097740/v1

**Published:** 2026-04-14

**Authors:** Anne C. Madeo, Kimberly A. Kaphingst, Melissa Yack, Erin D. Bouldin, Chelsey R. Schlechter, Sarah Dallas, Gwenndolyn Porter, Jennie L. Hill

**Affiliations:** University of Utah; University of Utah; University of Utah; University of Utah; University of Utah; University of Utah; Gwenn Porter Consulting; University of Utah

**Keywords:** Humans, Implementation Science, Rural Population, Genetic Testing, Qualitative Research, Idaho, Montana, Nevada, Wyoming, Utah

## Abstract

**BACKGROUND:**

Genetic testing is a cornerstone of precision medicine, yet evidence on rural genetic testing use is limited. Reduced access to specialty services and geographic barriers in rural settings may influence how rural residents experience and use genetic testing. Understanding contextual determinants and pre-implementation outcomes of clinician-ordered genetic testing is critical for enhancing access to precision medicine.

**METHODS:**

We conducted semi-structured interviews with rural residents in the Mountain West region of the United States who had discussed reproductive genetic carrier screening or disease risk genetic testing with a clinician. Interviews were thematically analyzed using a hybrid deductive–inductive approach informed by the Practical, Robust Implementation and Sustainability Model and the Implementation Outcomes Framework, with a focus on contextual determinants and the implementation outcomes acceptability and appropriateness.

**RESULTS:**

Participants were 30 predominantly college-educated non-Hispanic White residents of rural counties in Idaho, Montana, Nevada, Wyoming or Utah who had discussed disease risk or reproductive carrier genetic testing with a clinician. Participants generally perceived genetic testing discussed with a clinician as acceptable and appropriate, particularly when testing reduced uncertainty, supported reproductive decision-making or family responsibility, and was perceived as actionable. Ease and convenience of testing, out-of-pocket costs, insurance coverage, emotional readiness, and alignment with personal values influenced acceptability. Appropriateness was closely linked to clinician communication and interpersonal practices, including how clinicians explained testing, framed its relevance, and engaged patients in decision-making. Barriers to genetic testing for rural residents included having to navigate geographic distance, fragmented referral processes, and limited local specialty capacity, often relying on referrals to metropolitan healthcare systems.

**CONCLUSIONS:**

Among rural residents who discussed genetic testing with a clinician, genetic testing was primarily perceived as acceptable and appropriate; however, access was patient driven in the context of constrained rural healthcare infrastructure. These findings suggest that rural inequities in the uptake of precision medicine tools like genetic testing may be driven less by patient attitudes and more by the organization, communication, and delivery of genetic services within rural health systems.

## BACKGROUND

Precision medicine has been defined as an approach to disease treatment and prevention that considers individual variability in genes, environment, and lifestyle [1]. There is a significant evidence base supporting changes in reproductive management, risk assessment, disease prevention, and treatment based on an identified pathogenic variant (PV) in a disease-related gene or a gene tested in the reproductive setting [2–8]. Thus, genetic testing is a critical component of precision medicine and clinical genetic testing is currently recommended for reproductive genetic carrier screening (RGCS) and for specific disease(s) [3, 8–10].

Although recent research suggests that disparities in healthcare access among rural populations [11] may not extend to genetic testing [12, 13], rural residents may experience barriers to obtaining genetic services that are unique to their settings.. There is little data on barriers identified by rural residents to their access to genetic testing. A systematic review of barriers to genetics *referral* that included studies from urban and rural areas in the U.S. and abroad suggests that in rural areas the following non-genetics-trained clinician-related factors were barriers to referral: a lack of knowledge about genetics and genetic conditions, a lack of awareness of genetic services, inadequate referral coordination, lack of genetic workforce in rural areas [14]. Despite these barriers to referral, professional society guidelines recommend that all pregnant individuals or those considering reproduction be offered RGCS, ideally prior to a pregnancy is initiated [3, 9, 15]. Similarly, multiple professional society guidelines recommend disease risk genetic testing (DRGT) for individuals with a personal or family health history that is suspicious for inherited adult-onset conditions, including cancer and coronary heart disease [4, 5, 8, 16–18]. It is estimated that 2–3% of the U.S. population harbors a PV in a gene responsible for Familial Hypercholesterolemia, *BRCA1/2* (the most common causes of Hereditary Breast and Ovarian Cancer) or Lynch syndrome (an inherited cancer syndrome, believed to be responsible for 2–4% of colorectal cancer) [19–28]. Identifying a PV responsible for one of these conditions allows for individualized screening and treatment recommendations [4, 5, 7, 8, 10, 29] therefore, the goal is to provide genetic consultation if a disease risk is identified based on personal or family history [8, 16]. Despite the potential benefits of receiving RGCS or DRGT, many individuals in the U.S. do not receive these tests [8, 24, 25, 30–34].

To better understand the contextual determinants of genetic testing and perceptions of its acceptability and appropriateness among rural residents of a Mountain West State (Idaho, Nevada, Montana, Wyoming or Utah) who discussed RGCS or DRGT with a clinician, the research team (comprised of the authors) interviewed rural residents about their experiences surrounding genetic testing.

## METHODS

We followed the COREQ (COnsolidated criteria for REporting Qualitative research) Checklist for reporting the study results [35].

### Ethics Review

This study was reviewed by the University of Utah Institutional Review Board (IRB_00176898) which deemed it exempt from documentation of consent. All participants received a consent cover letter. Although consent to participate was considered implied by an individual’s participation in the interview, prior to the interview informed consent was obtained from all participants, Participants affirmed their willingness to participate and have their interview recorded.

### Conceptual model

**Figure 1:**
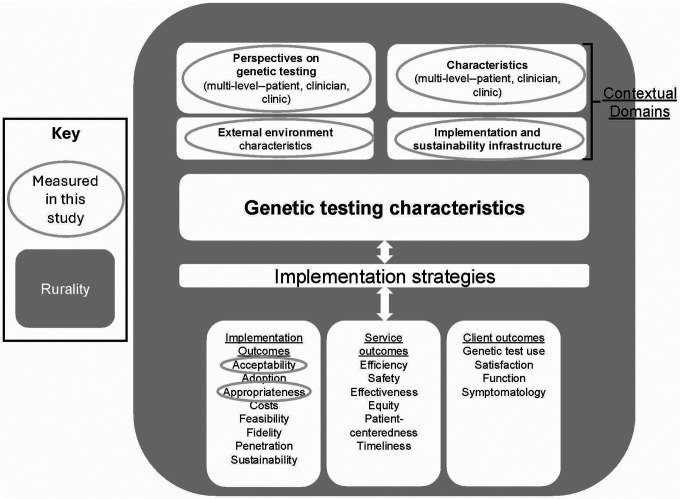
Conceptual model that informed study, based on the Practical, Robust Implementation and Sustainability Model [36] and the Implementation Outcomes Framework [37].

To guide the identification of key factors in genetic medicine implementation, this study was informed by a theoretical model that integrated the Practical, Robust Implementation and Sustainability Model (PRISM) [36] and the Implementation Outcomes Framework (IOF) [37] (Figure 1). PRISM was selected for its pragmatic focus on understanding the contextual factors that affect the effectiveness of healthcare interventions in real-world settings and its use in planning genetic medicine implementation [38]. It includes the following multilevel contextual domains relevant to program implementation throughout all stages: (1) Intervention: Patient Perspective, (2) Characteristics: Patients, (3) Implementation and Sustainability Infrastructure, and (4) External Environment. IOF was selected for its focus on implementation outcomes that are crucial for understanding implementation effectiveness and for suggesting that these outcomes could be targets for implementation strategies [39]. In this study, we considered the implementation outcomes acceptability (“the perception among implementation stakeholders that a given treatment, service, practice, or innovation is agreeable, palatable, or satisfactory” [37]) and appropriateness (“the perceived fit, relevance, or compatibility of the innovation or evidence based practice for a given practice setting, provider, or consumer; and/or perceived fit of the innovation to address a particular issue or problem” [37]), as they are pre-implementation outcomes that can be measured with semi-structured interviews at the individual level of analysis [37].

### Study recruitment and sample size.

Participants were recruited from multiple sources. The study was posted on a public studies page hosted by the University of Utah Office of Research Participant Advocacy. Recruitment flyers were sent to rural clinics to post at their clinics and to Primary Health Care Associations in Idaho, Nevada, Montana, Wyoming and Utah to distribute to member organizations. The Community Collaboration and Engagement Team (CCET) at the University of Utah contributed to this project by facilitating recruitment of rural residents who discussed RGCS with a clinician. Individuals were identified through direct contact with the Montana Medical Examiners Board of licensees and the Nevada State Board of Nursing licensees, review of charts screened with the University of Utah Health electronic health record and the University of Utah Huntsman Cancer Institute’s Inherited Cancer Research Shared Resource, and direct referrals. Our *a priori* sample size was 15 individuals per genetic testing type (i.e. DRGT and RGCS), based on typical sample sizes sufficient to achieve thematic saturation in investigations of experiences and phenomena [40–42].

### Eligibility

Eligibility criteria for both groups included living in a rural county (defined as 2023 Rural Urban Continuum (RUC) [43] codes 4 – 9) in one of five Mountain West states (Idaho, Montana, Nevada, Wyoming, and Utah), having discussed RCGS or DRGT with a clinician, and feeling comfortable participating in an interview conducted entirely in English using a video conference service. Individuals could present to the interview alone or accompanied by whomever they wanted and communicate with the interviewer from any location.

Individuals who were eligible for the RGCS interviews if they were aged 18 – 50. The upper age limit was established as an eligibility criterion to include individuals who were pregnant after the American College of Obstetricians and Gynecologists and the American College of Medical Genetics and Genomics jointly recommended offering population-based reproductive cystic fibrosis carrier testing in 2001 [44] and because this was the age range we established for our quantitative analysis of RGCS in HINTS data [13]. Individuals who discussed RCGS and were identified through the University of Utah Health electronic health record must have been seen by a University of Utah Health OB/GYN clinician in an outpatient visit within the previous 36 months and been assigned female at birth. Sex assigned at birth was established for individuals identified through the University of Utah Health electronic health record because professional guidelines recommend offering RGCS either in serial or parallel to a reproductive pair [3, 9, 45] and previous research has suggested that men and women may consider different factors when deciding whether to pursue RGCS [46]. In addition, individuals assigned female at birth were expected to be seen by clinicians in the University of Utah Obstetrics and Gynecology department. Individuals were eligible for DRGT interviews if they were aged 18 – 75 years old.

### Screening potential participants

Potential participants who learned of the study via a flyer were invited to complete a REDCap (Research Electronic Data Capture) eligibility survey, hosted at the University of Utah [47–49]. Potential participants who were recruited through other sources received ≤5 contacts, initially via email or text, inviting them to complete the REDCap eligibility survey and provided a link to a consent cover letter. All individuals who completed the REDCap survey were informed whether a preliminary assessment by an investigator indicated they were likely eligible. Individuals who had no known previous contact with an investigator were provided a link to a consent cover letter. If the preliminary assessment indicated eligibility requirements were fulfilled, participants were invited to schedule a 30-minute interview via Zoom^®^ [50].

**Figure 2:**
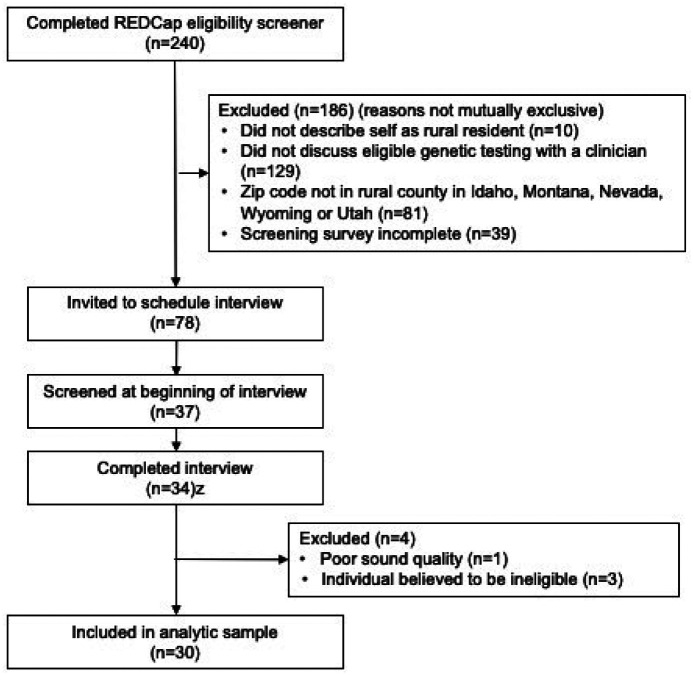
Participant selection.

Given the broad public-facing recruitment strategy and based on published recommendations [51, 52], a two-step screening process was used to reduce the likelihood of participation by non-humans (i.e., bots), people outside the geographic area of interest or other ineligible participants. Individuals who scheduled and presented for an interview were further screened before the interview began with a standardized set of questions to confirm details in found in the initial REDCap screen. Individuals who could not answer at least five screening questions concordant with their responses in REDCap, were informed that the research team would follow up with them if additional participants were needed and further interview questions were not pursued. Individuals whose responses to the second screen were concordant with their REDCap eligibility survey were interviewed. The research team discussed and assessed the eligibility of participants who were suspected of being ineligible (e.g. individuals who declined invitations to turn on their camera, whose sound quality/connection to the interview was poor, and/or who were unable to accurately report a recruitment venue with which the research team was familiar [51, 52], and/or they did not appear eligible on the basis of their responses to interview questions) and discarded interview data from individuals determined to be likely ineligible (Figure 2). All participants who completed an interview received $75.

### Interviews

The authors developed semi-structured interview guides tailored to each eligibility group with core questions and optional probes (Supplementary Material). Interview guides drew on the PRISM and IOF models as well as our previous research and were designed to elicit respondent feedback across PRISM domains, acceptability and appropriateness [13]. Guides were pilot tested with two eligible interviewees whose data were included in analysis, then minorly modified for flow and understanding. Interviews were conducted over 11 months between January and November of 2025 by ACM, a cisgender White woman doctoral student and board-certified genetic counselor who lives in an urban area of a Mountain West state that consists primarily of rural counties. She completed coursework and training in qualitative methods and semi-structured interviewing. She had no prior relationship with any participants and answered participants’ questions about her interest in the topic. She took notes during and/or after interviews.

Interviews were audio and video recorded. Audio recordings were professionally transcribed and deidentified. The interviewer was alone in a private location in her home throughout all interviews. Interviews ranged from 16 to 46 minutes (mean 27 minutes). Participants were not provided with a copy of their transcripts.

Recruitment and interviewing continued until the *a priori* sample size was achieved. During analysis, we observed that this sample size was sufficient to reach thematic saturation—defined as the point at which no new themes or conceptual insights emerged [53]. This was assessed by reviewing coded segments from transcripts to determine if codes mapped consistently onto existing themes and whether new emergent codes were necessary to explain participants’ experiences with genetic testing. No participants were interviewed more than once.

### Analysis

We conducted a thematic analysis using a hybrid coding approach consistent with Boyatzis [54] and Crabtree and Miller [55]. We began with deductive codes based on an *a priori* codebook informed by the conceptual model, and added inductive codes to capture themes that emerged from the data.

In September 2025, two trained research assistants (RAs), a senior qualitative researcher (JLH), and the interviewer, (ACM) used Microsoft Excel [56] to manage and apply the initial deductive codebook to two transcripts and inductively identify emergent codes, which were defined and added to the codebook. Following the initial codebook development, a reconciliation meeting was held to align codebook definitions, with consensus defined as agreement by all participants. Transcripts were subsequently independently coded by two RAs. The research team collaboratively revised the codebook and transcript coding in regular meetings. During the coding process, the research team changed; incoming team members were trained on the existing codebook before coding transcripts. All transcripts were coded according to the finalized codebook (Supplementary Material). ACM reviewed and reconciled coded segments of transcripts, coding discrepancies that could not be addressed without input from RAs were reconciled based on their feedback. Participants were not asked to provide feedback on findings.

Themes were identified by sorting and examining text segments with shared codes and by making connections among them; they were then reviewed by the analytic team [55]. This was an iterative process of immersion, crystallization/connecting, and corroboration [57] undertaken by ACM, JLH, GP, and SD. Global themes were generated to reflect cross-cutting insights across eligibility groups. Summary tables with illustrative quotes were reviewed and discussed by the research team. Rigor was supported throughout coding and analysis by researchers recording analytic memos and staying close to the data.

## RESULTS

Sociodemographic data on interviewed eligible participants are presented in Table 1. Participants (n=30) were predominantly college educated, non-Hispanic White individuals who had both a personal and a family history of a health condition they believed was inherited and had received genetic testing. Gender and age helped define eligibility. Tables 2–10 summarize the qualitative themes identified for each of the PRISM constructs and the IOF outcomes Acceptability and Appropriateness, with representative supporting quotes. Themes from PRISM domains are presented before IOF outcomes. PRISM domains are presented here in the order “Perspectives” (participant and clinician levels), “Characteristics” (participant, clinician and organization levels), External Environment and Implementation and Sustainability Infrastructure. Themes in each domain overlap, as the defining characteristic of the code applied was the way that the person described a segment.

### Perspectives on genetic testing

#### Participant

Cross-cutting themes from participants’ perspectives on genetic testing, how they understand, value and experience it, (Table 2) indicate that (1) participants frequently believe genetic testing offers clarity and reassurance even when its clinical scope is limited; (2) participants consider genetic testing a moral and relational responsibility to protect family members across generations. (3) clinician framing and relationships with participants inform participants’ understanding of genetic testing, and (4) the perceived value of genetic testing is relative to its cost and insurance coverage. One theme was unique to the RGCS eligibility group: Genetic testing is a practical tool to anticipate future decisions. These themes reflect not only what genetic testing is perceived to offer, but also how multiple contexts (e.g. perceived familial responsibilities, testing cost and insurance coverage) influenced its perceived utility. Together, the themes highlight that, for participants, genetic testing occurs within a web of familial and clinical relationships that is combined with a desire for certainty and meaning, all of which are balanced against genetic testing’s cost and insurance coverage. These perspectives inform participants perspectives on its acceptability and perceived fit in their lives, more than the technical details that supported it.

#### Participant understanding of clinicians’ perspectives

Participants described clinicians as helping them understand the clinical actionability and importance of genetic testing. Their clinicians helped participants understand the clinical actionability and importance of genetic testing. Participants interpreted clinicians’ engagement and recommendations as indicators of the clinical importance of genetic testing (Table 3). When clinicians actively raised the topic of, or endorsed, genetic testing, participants inferred that it was meaningful, whereas limited enthusiasm or follow-through by clinicians signaled lower priority. Participants frequently linked these legitimacy judgments to clinicians’ apparent knowledge or confidence (i.e., perceived uncertainty translated into weaker endorsement.) As one participant noted, “[clinician] didn’t seem like they knew very much… so it was not something they really pushed” (R-10; Table 3).

#### Organization

No quotes from participants were coded to the PRISM domain Organization level Perspectives on genetic testing.

### Characteristics

#### Participant

Participants who discussed DRGT with a clinician frequently drew on family health history (Table 4) to interpret their own genetic risk, using patterns of illness among relatives as evidence of an inherited vulnerability to disease. Across disease conditions, participants described having multiple affected family members as heightening concern and motivating their interest in genetic testing. No quotes from participants who discussed RGCS were coded as Participant Characteristics

#### Clinician

Participants who described clinician characteristics (Table 5) indicated that their characteristics affected how they interacted with genetic testing, emphasizing the quality of clinician–patient interactions as determinants of trust, understanding, and perceived quality of care. Clear communication and empathy were described as evidence of good quality care. In contrast, impersonal or fragmented interactions were perceived as making access to genetic testing more difficult and uncertain. No quotes from participants who discussed RGCS were coded to this domain.

#### Organization

Participants described features of rural health systems (Table 6) as influencing how healthcare was delivered to them and they experienced it. While not all their comments pertained to genetic services, many themes that reference specialty care services may apply to genetic services. For example, limited organizational capacity or outdated services constrained testing in some settings. Fragmented referral and scheduling processes delayed testing and provided sufficient friction that it deterred some participants from obtaining it. In contrast, organizational integration of genetic testing facilitated its uptake (e.g. When services were embedded within existing care through point-of-care sample collection or coordinated visits.) At the same time, communication and follow-up practices by organizations and clinics influenced whether participants felt confused or reassured.

### External Environment

The External Environment is the broader context (beyond a clinic) where genetic testing is delivered. Participants described multiple features of the external environment (many of which are unique to rurality) that make access to genetic testing challenging (Table 7). The themes that arose in these quotes included (1) geographic isolation and travel burden; (2) limited local capacity led participants to rely on distant healthcare systems, and (3) Insurance and billing complexity added layers of access difficulty within rural healthcare systems.

### Implementation and Sustainability Infrastructure

Participants who discussed RGCS with a clinician described genetic testing as integrated into routine prenatal care through standardized screening workflows, clinician-supported decision-making, and established laboratory partnerships (Table 8). This embedded implementation reduced reliance on individual clinician/patient initiative or local capacity and supported the continued offering of genetic testing through normalization/routinization, consistency in processes and institutional capacity. No quotes from participants who discussed DRGT were coded in this domain.

### Acceptability

Rural residents described genetic testing acceptability (Table 9) as related to its ability to provide information or reduced uncertainty, support family responsibility, and offer information perceived as actionable for future health or reproductive decisions. Acceptability was strongly influenced by factors such as ease and convenience of testing, out-of-pocket cost and insurance coverage, and clinicians’ framing and recommendations, which were perceived as legitimizing testing. At the same time, emotional readiness, mental health burden, and personal values—such as beliefs about autonomy or fate—introduced important variation in how individuals perceived the acceptability of genetic testing.

### Appropriateness

Participants described the appropriateness of genetic testing (Table 10) as shaped by clinician communication and interpersonal practices, particularly how clinicians explained the purpose of testing, framed its relevance, and engaged patients in decision-making. Clear, empathetic, and proactive communication, expectations regarding what genetic testing may reveal, and a desire to protect one’s family helped rural residents perceive that genetic testing was clinically appropriate for their situation. In contrast, impersonal or fragmented interactions with clinicians contributed to uncertainty about whether testing was warranted.

## DISCUSSION

In this qualitative study of rural residents in the Mountain West who had discussed RGCS or DRGT with a clinician, we identified contextual factors that influence how participants access and experience genetic testing. Guided by PRISM and IOF, our findings suggest that genetic testing discussed with a clinician was generally perceived by participants as acceptable and appropriate. However, access often depended on participants’ ability to navigate geographic, clinical, and logistical barriers.

Across both testing types, participants described genetic testing as valuable for reducing uncertainty, informing family members, and supporting future planning. Similar motivations have been reported in prior studies, in which individuals often experience reassurance and changes in perceived risk following genetic testing, including when results are ambiguous and do not lead to changes in clinical management [58, 59]. In this study, the value of genetic testing was commonly framed in psychosocial and informational terms, which emphasizes the importance of patient-perceived benefits (i.e., personal utility [60]).

Participants’ assessments of whether genetic testing was appropriate were closely tied to clinician communication and their relationships with clinicians. Clinicians’ framing and engagement strongly influenced participants’ interpretations of the relevance and legitimacy of genetic testing. This finding is consistent with prior work demonstrating that clinician referral practices, knowledge, and attitudes play a central role in patient access to genetic services, particularly outside of specialty genetics settings [14, 61]. In rural contexts and other settings where access to specialty services is limited, non-genetics-trained clinicians may play a particularly influential role in shaping patients’ understanding and decision-making regarding genetic testing [62].

A central finding of this study is that rural healthcare systems appear to shift much of the work required to obtain genetic testing to patients. Participants commonly described traveling long distances, coordinating care across organizations, and managing referral delays or fragmented communication.

This pattern is consistent with the broader rural health services literature, which documents that limited local healthcare capacity often results in greater patient burden in accessing specialty care, including cancer services and advanced diagnostics [63, 64]. From a health services perspective, access to genetic testing in rural settings may be maintained through patient effort rather than through locally available infrastructure. However, this transfer of responsibility may not be unique to rural settings, as underserved individuals (e.g. uninsured [65, 66]) in other settings (e.g. Federally Qualified Health Centers [67]) may experience similar transfers of responsibility.

These findings help contextualize prior quantitative analyses, such as our own work using nationally representative survey data, which found no, or only marginally significant, rural–urban differences in the prevalence of genetic testing among rural residents [12, 13, 68]. Similar estimates of genetic testing prevalence may obscure important differences in how services are delivered and experienced. Previous research has noted that comparable utilization rates can mask disparities in travel burden, timeliness, and care coordination, particularly among rural populations [69, 70]. Qualitative data from this study suggest that rural residents who obtain genetic testing may do so by compensating for limited local capacity, potentially concealing differences in the conditions under which care is accessed. Importantly, non-rural residents may also experience challenges obtaining genetic testing that may also be due to limited access to specialty care [71], although these barriers may exist even if there are increases in genetics-trained specialists available [72].

Differences between RGCS and DRGT further highlight the importance of the implementation context. Reproductive carrier screening was often described as integrated into routine prenatal care and supported by standardized workflows The fact that offering RGCS is endorsed by the American College of Obstetricians and Gynecologists [9] may facilitate RGCS incorporation into routine prenatal care. Prior studies have identified barriers and facilitators to integrating genetic services into general clinical practice, including clinician knowledge, referral pathways, and service structure [73, 74]. In contrast to RGCS, DRGT was more variable in its incorporation into routine care. DRGT depended on clinician knowledge, referral processes, and follow-up, making it more sensitive to organizational and workforce constraints that have been well documented in rural healthcare settings [61, 75], although our research suggests that these barriers do not necessarily prevent rural residents from obtaining genetic testing [13]. However, there may be context specific barriers that facilitate genetic testing uptake among certain populations (e.g. individuals with insurance) that may not have been captured in this analysis.

These findings have implications for the delivery of genetic services in rural regions. Efforts to improve access should focus on reducing the logistical and coordination burdens placed on patients, rather than solely increasing awareness among rural residents or clinicians. Prior work suggests that strategies such as population-based cancer family history screening, strengthened referral pathways, expanded use of telehealth-supported genetics services, greater integration of testing processes into non-specialty care settings may help mitigate geographic disparities [76–78]. Similar solutions have been proposed to integrate medical genetics into primary care broadly in underserved populations [79].

Previous research has suggested that the barriers to genetic testing experienced by non-rural populations include clinician knowledge gaps about genetics or genetic testing [74, 80]. In this study, residents perceived clinicians as having knowledge gaps that contributed to the way residents perceived their conversations with clinicians and the importance that they felt their clinicians placed on genetic testing and subsequently, the appropriateness of genetic testing for their care. Clinicians have also described uncertainty with how to make a referral to genetics [14, 81] and likely do not refer all eligible patients to genetics [82, 83]. Although we do not describe challenges clinicians experienced with making referrals or their knowledge of referral pathways, based on resident descriptions one of the organizational characteristics that functioned as a barrier to obtaining genetic services was fragmented referral pathways. This has been previously reported as a barrier to genetics referral, including in rural areas [14]. Although the cancer family history remains a mainstay of assessing eligibility for genetic services [4, 5], there are ample data that primary care clinicians in urban [84] and rural [61] areas do not routinely collect family health history information and in urban areas there are differences in who within a family knows family health history information [85, 86] which likely extend to rural populations.

Perhaps because individuals who felt comfortable talking with an interviewer were those who responded, rural residents did not mention clinician communication as a barrier to receiving genetic testing, although clinician communication has come up in previous research as a barrier to patients receiving genetic services [87, 88]. However, rural residents reported that clinicians who communicated clearly and with empathy facilitated their ability to navigate the challenges they may have faced obtaining genetic services (e.g. limited local specialist availability and complex referral processes in local and referral care settings) and clinician support of genetic testing (through providing meaning and legitimacy to testing) supported their test uptake.

This study has limitations. Participants had discussed genetic testing with a clinician; therefore, our findings do not capture barriers experienced by individuals who never reach this point (e.g., many individuals who have not previously heard of genetic testing). Differences in themes among participant subgroups were not explored as it was outside the scope of the present study. The sample was limited to English-speaking participants in the Mountain West, which may limit transferability to other rural regions (particularly those that do not share the Mountain West’s geographic [63, 89] and social features [90]) or to non-English-speaking populations. However, as with all qualitative research, this study aimed to generate an in-depth understanding of genetic services in Mountain West rural counties rather than generate generalizable data.

## CONCLUSION

Rural residents who discussed clinician-ordered genetic testing with a clinician generally viewed it as acceptable and appropriate, but access was often achieved through additional resident effort in limited local capacity. These findings suggest that low genetic testing rates among rural populations may be driven less by patient attitudes and more by the organization and delivery of genetic services within rural health systems.

## Supplementary Material

Supplementary Files

This is a list of supplementary files associated with this preprint. Click to download.

- Aim2DRAFTtablesv27.docx
- InterviewGuideResidentcarrier.docx
- Codebook.xlsx

## Data Availability

The datasets generated and analyzed during the current study are not publicly available due to concerns regarding the privacy of individuals who participated in the study but are available from the corresponding author on reasonable request.

## Abbreviations

COREQ: COnsolidated criteria for REporting Qualitative research
DRGT: Disease Risk Genetic Testing
IOF: Implementation Outcomes Framework
PRISM: Practical, Robust Implementation and Sustainability Model
PV: Pathogenic Variant
RA: Research Assistant
RGCS: Reproductive Genetic Carrier Screening
